# Cardiac dysfunction and remodeling regulated by anti-angiogenic environment in patients with preeclampsia: the ANGIOCOR prospective cohort study protocol

**DOI:** 10.1186/s12884-021-04263-w

**Published:** 2021-12-08

**Authors:** Johana Ullmo, Monica Cruz-Lemini, Olga Sánchez-García, Lidia Bos-Real, Patricia Fernandez De La Llama, Francesca Calero, Carla Domínguez-Gallardo, Carmen Garrido-Gimenez, Cristina Trilla, Francesc Carreras-Costa, Alessandro Sionis, Josefina Mora, Álvaro García-Osuna, Jordi Ordoñez-Llanos, Elisa Llurba

**Affiliations:** 1 Obstetrics and Gynecology Department, Santa Creu i Sant Pau University Hospital & Universitat Autònoma, Barcelona, Spain; 2 Woman and Perinatal Health Research Group, Sant Pau Biomedical Research Institute (IIB-Sant Pau), Sant Pau University Hospital, Barcelona, Spain; 3 Maternal and Child Health and Development Network (SAMID), RD16/0022/0015, Instituto de Salud Carlos III, Madrid, Spain; 4 Cardiology Department, Santa Creu i Sant Pau University Hospital, Barcelona, Spain; 5 Nephrology Department, Hypertension and Prevention of Kidney Damage Unit, Fundació Puigvert, Barcelona, Spain; 6 Biochemistry Department, Santa Creu i Sant Pau University Hospital & Universitat Autònoma, Barcelona, Spain; 7 Fundació per la Bioquímica i la Patología Molecular, Biochemistry Department, Santa Creu i Sant Pau University Hospital & Universitat Autònoma, Barcelona, Spain

**Keywords:** Preeclampsia, Cardiac dysfunction, Cardiac remodeling, sFlt-1, PlGF, Angiogenic factors, Cardiac biomarkers, Cardiovascular risk

## Abstract

**Background:**

Cardiovascular diseases (CVD) are cause of increased morbidity and mortality in spite of advances for diagnosis and treatment. Changes during pregnancy affect importantly the maternal CV system. Pregnant women that develop preeclampsia (PE) have higher risk (up to 4 times) of clinical CVD in the short- and long-term. Predominance of an anti-angiogenic environment during pregnancy is known as main cause of PE, but its relationship with CV complications is still under research. We hypothesize that angiogenic factors are associated to maternal cardiac dysfunction/remodeling and that these may be detected by new cardiac biomarkers and maternal echocardiography.

**Methods:**

Prospective cohort study of pregnant women with high-risk of PE in first trimester screening, established diagnosis of PE during gestation, and healthy pregnant women (total intended sample size *n* = 440). Placental biochemical and biophysical cardiovascular markers will be assessed in the first and third trimesters of pregnancy, along with maternal echocardiographic parameters. Fetal cardiac function at third trimester of pregnancy will be also evaluated and correlated with maternal variables. Maternal cardiac function assessment will be determined 12 months after delivery, and correlation with CV and PE risk variables obtained during pregnancy will be evaluated.

**Discussion:**

The study will contribute to characterize the relationship between anti-angiogenic environment and maternal CV dysfunction/remodeling, during and after pregnancy, as well as its impact on future CVD risk in patients with PE. The ultimate goal is to improve CV health of women with high-risk or previous PE, and thus, reduce the burden of the disease.

**Trial registration:**

NCT04162236

## Background

Cardiovascular diseases (CVD) are cause of increased morbidity and mortality in spite of advances for diagnosis and treatment. Manifestations of CVD, risk factors and treatment are different in women than in men; women are affected by traditional CVD risk factors, but also by gender-specific ones, such as polycystic ovarian syndrome or pregnancy-associated disorders such as preeclampsia (PE) [1]. In 2011, the American Heart Association (AHA) guideline for preventing CVD in women included PE, gestational hypertension, diabetes mellitus, and systemic autoimmune collagen-vascular disease (i.e., lupus or rheumatoid arthritis) as factors associated with increased risk of CVD [2]. Later, in 2018 and 2019, the AHA/ACC Multisociety Guideline on the Management of Blood Cholesterol [3]; and the American College of Cardiology (ACC)/AHA guidelines [4] introduced the concept of women-specific factors to consider for risk, diagnosis and treatment of CVD; these factors now included PE.

One of the most prevalent complications of pregnancy is PE. Epidemiological studies have shown that PE is associated with development of CV risk factors, hypertension, myocardial infarction, stroke, renal complications, vascular and metabolic dysfunction during pregnancy and in later years. Pregnant women that develop preeclampsia (PE) have higher risk (up to 4 times) of clinical CVD in the short- and long-term [5, 6]. When PE is accompanied by preterm birth and/or small for gestational age, the adjusted risk for CVD is increased by 45% for the next 5 years post-partum [7]; risk comparable to that of smoking. Therefore, as history of PE has recently been incorporated by the aforementioned societies as an independent risk factor for CVD [8], follow-up starting within the fourth decade of life is recommended by some authors [9]. All these facts highlight the need to prevent and diagnose PE during pregnancy, and follow-up these women after delivery.

Changes during pregnancy affect importantly the maternal CV system, in order to maintain adequate fetal blood perfusion; maternal cardiac output and heart rate increase, while blood pressure and vascular resistances decrease [10]. In PE, CV adaptation in pregnancy is abnormal, exceeding physiological levels, which occurs more frequently if there is a hypertensive disorder before pregnancy. In fact, CV findings in PE resemble those due to cardiac diastolic dysfunction; i.e., improper left ventricle (LV) relaxation, increased LV chamber stiffness, and increased cardiac filling pressures [11], all of which can be detected by echocardiography.

PE is also characterized by endothelial dysfunction, which results from an imbalance between maternal circulating angiogenic factors, like pro-angiogenic placental growth factor (PlGF), and anti-angiogenic factors such as soluble fms-like tyrosine kinase 1 (sFlt-1) [12]. Placental expression and maternal circulating concentrations of sFlt-1 are increased in PE [13], whereas PlGF is decreased. This occurs due to a combination of decreased expression because of poor placentation, and reduced free PlGF due to sFlt-1 binding [14]. This leads to an increase in the sFlt-1/PlGF ratio, which has been described as better predictor for PE severity than either marker alone [15]. Recently, our group reported an association between angiogenic factors during pregnancy and CV disease 12 years post-partum, suggesting angiogenic factors are important players in PE-associated CV risk later in life [16].

In addition to maternal CV effects, fetal cardiac programming during pregnancy and its relationship with angiogenic environment has not been previously assessed. Fetal programming refers to the impact on fetal health due to intrauterine environment [17]. It occurs when the fetal environment is modified by an adverse condition in a critical moment of development. Pregnancy complications such as PE and intrauterine growth restriction are associated with fetal programming [18, 19]. However, little is known on the role of angiogenic biomarkers for early detection of fetal cardiac abnormalities and programming in PE pregnancies.

Multiple biomarkers have been assessed to evaluate CVD risk or myocardial damage in PE. Arginine vasopressin (AVP) has been shown to reproduce main maternal and fetal PE features in animal models [20] and some studies propose its more stable metabolite, copeptin, as an early predictor of PE [21]. Thus, AVP concentration is estimated measuring copeptin. Cardiac troponins (cTn) are isoforms solely produced by myocardial cells. When measured by high-sensitivity (hs) methods, cTn can detect small amounts released in myocardial ischemia. Few studies have analyzed hs-cTn in PE [22]; thus, it remains to be determined whether hs-cTn could detect minor myocardial damage in PE women. Natriuretic peptides are biomarkers of cardiac volume overload, vascular hypertension and myocardial elongation. All these conditions are observed in PE, and particularly those of the B-family (B-type natriuretic peptide, BNP), have been analyzed in normal and hypertensive pregnancies and found to be biomarkers for CV alterations in PE [23]. BNP is synthetized as a prehormone (pro-BNP), which is cleaved to an N-terminal fragment (NT-proBNP), and both are associated with hemodynamic stress and heart failure.

We hypothesize that an association exists between the anti-angiogenic environment and cardiac dysfunction/remodeling in women at risk for PE during pregnancy and their offspring, and that this relationship could determine an increased CV risk later in life.

## Aims

### Primary aim

- To determine if a relationship exists between angiogenic environment during pregnancy and the risk of maternal cardiovascular dysfunction and remodeling.

### Secondary aims

- To prospectively assess both biochemical and biophysical CV markers in a cohort of patients with risk for PE and in healthy pregnant women, in the first and third trimesters of pregnancy.
- To determine correlation between angiogenic (PlGF) and anti-angiogenic (sFlt-1) factors in maternal serum, and biochemical and imaging markers of cardiac dysfunction and remodeling in patients with established PE.
- To evaluate the relationship between placental and maternal cardiac biomarkers and fetal cardiac function in the third trimester of pregnancy.
- To determine cardiac dysfunction and metabolic status 12 months after delivery in women with PE, and its correlation with CV and PE risk variables obtained during pregnancy.
- To investigate angiogenesis and CV-related genes in placental samples of women with PE and controls, by microarray and polymerase chain reaction (PCR) validation.
- To develop a combined model for prediction of cardiovascular risk in PE, that includes placental and cardiac biomarkers, clinical and echocardiographic variables.

## Methods

### Trial registration

https://clinicaltrials.gov/NCT04162236 .

### Study design

Prospective cohort study which will include the following patients: pregnant women with high-risk of PE in first trimester screening, established diagnosis of PE during gestation, and healthy pregnant women (controls).

### Setting/location

Santa Creu i Sant Pau University Hospital, Universitat Autònoma de Barcelona, Barcelona, Spain.

### Inclusion criteria

Women between 18 and 45 years of age, with singleton pregnancies and high-risk for PE according to our first trimester screening protocol [24], which includes registration/measurement of maternal risk factors, blood pressure (BP), pregnancy-associated plasma protein A (PAPP-A) and mean uterine artery pulsatility index (UtA-PI), at 11^+ 0^ to 13^+ 6^ weeks of gestation. The estimated sample size for the high-risk group is 280 patients, who will be divided according to the subsequent development of PE:

- True PE: women from the high-risk group who develop PE (estimated *n* = 40)
- False PE: women from the high-risk group who do not develop PE (estimated *n* = 240)

An additional group of healthy pregnant women with first trimester low-risk screening for PE will be recruited (*n* = 100).

Additionally, women with established diagnosis of PE (*n* = 60) that were not participating previously in the study, will be included at time of diagnosis. The local research coordinator and/or staff will identify eligible women, invite them to participate, provide written and oral information, and obtain the written informed consent.

### Exclusion criteria

Pregnancies with previous maternal conditions such as thrombophilia, type-1 diabetes, hyperthyroidism, any renal disease, severe maternal illness, cytomegalovirus or toxoplasmosis infection, maternal human immunodeficiency virus infection, previous venous or arterial thrombotic events, alcohol or illicit drug use, as well as multiple pregnancies, major fetal anomaly or chromosomal anomalies.

### Procedures and interventions

**Fig. 1:**
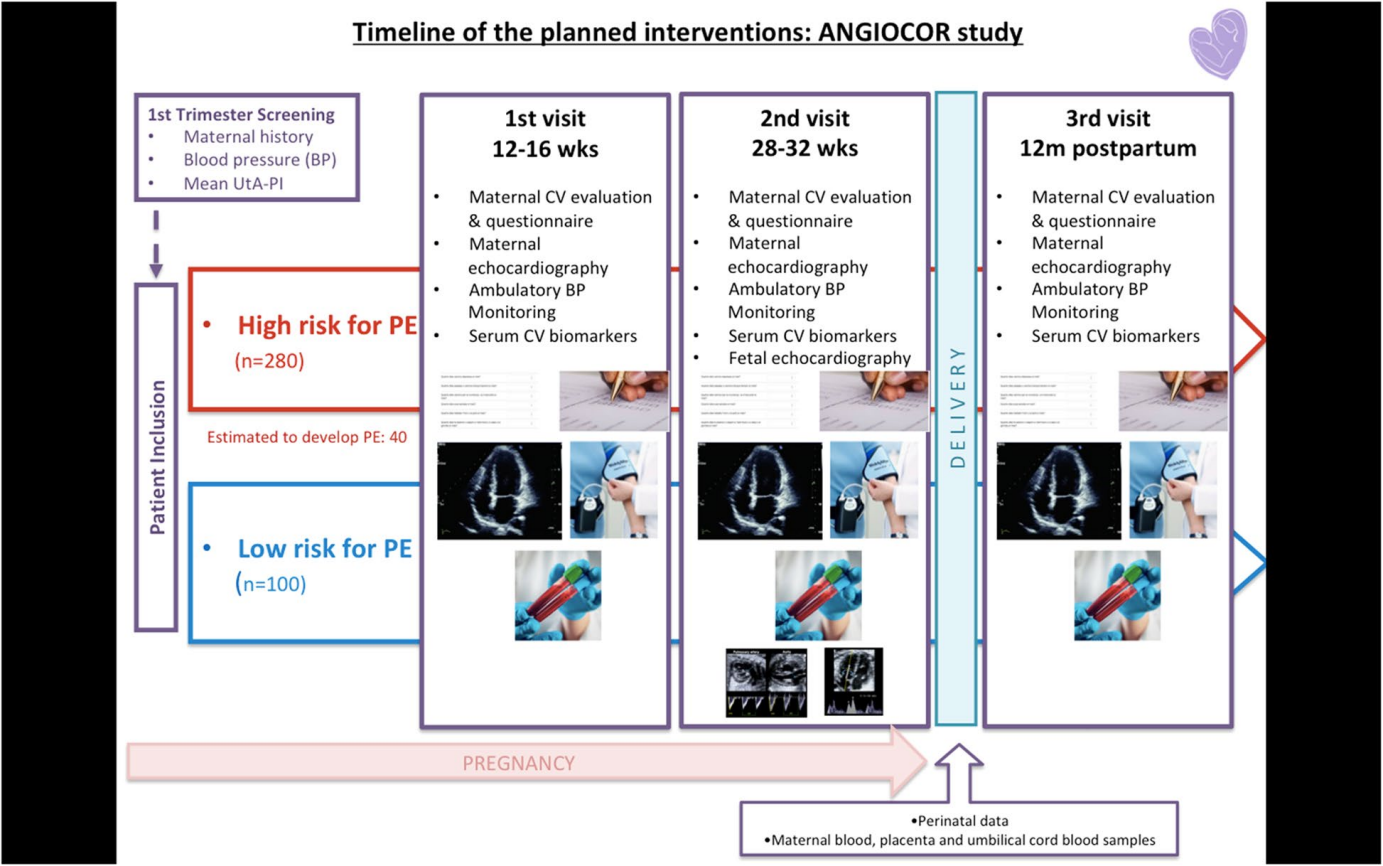
Timeline of the planned interventions: ANGIOCOR study. PE, preeclampsia; CV, cardiovascular; BP, blood pressure; UtA-PI, uterine artery pulsatility index

Patients will be invited to participate after first trimester screening (11-13^+ 6^ weeks); once they have consented to the study, a maternal echocardiographic evaluation will be performed, and blood and urine samples will be obtained. Patients will be scheduled at third trimester (28-32 weeks of pregnancy) for a second maternal echocardiographic evaluation, a fetal functional echocardiographic evaluation and a second blood sample. If a patient develops PE before the scheduled evaluations, all studies will be conducted before delivery. At birth, umbilical cord blood, maternal blood and placental samples will be obtained for evaluation of biomarkers. Finally, a maternal echocardiographic evaluation will be scheduled 12 months after delivery, with final blood and urine samples for biomarker analysis. Figure 1 shows the timeline for the planned interventions.

#### Maternal echocardiography

Maternal echocardiographic evaluation will be performed at first trimester, 28-32 weeks’ gestation and 12 months postpartum, by a Cardiologist specialized in cardiac imaging, according to the usual standard protocol [25]. High-resolution images will be acquired with Affinity 70G (Philips, Andover, MA, USA) ultrasound machine, with post-processing analysis with a dedicated cardiovascular software, including myocardial strain analysis by 2D speckle-tracking (Philips AutoStrain LV). The following parameters will be evaluated: LV dimensions (end-diastolic diameter (EDD), end-systolic diameter (ESD), end-diastolic volume (EDV), end-systolic volume (ESV)) and thickness (interventricular septum (IVS) and posterior wall (PW)), left ventricular ejection fraction (EF) obtained by Teicholz and Simpson methods, LV myocardial deformation (strain) parameters, cardiac output, left atrial volume, Doppler transmitral flow analysis including tissue Doppler imaging (TDI) and right heart evaluation including ventricular diameters, tricuspid annular ring systolic excursion (TAPSE) and TDI of the tricuspid lateral annulus.

#### Fetal echocardiography

Fetal echocardiographic examination will be performed at 3rd trimester of gestation according to the International Society of Ultrasound in Obstetrics and Gynecology guideline [26]. Briefly, cardiac axis and situs, pericardial effusions, ventricular morphology, veno-atrial, atrioventricular and ventricle-arterial connections, size and relationships of left and right ventricular outflow tracts, ductal and aortic arches, atrial size, interventricular septum (IVS) and flow through atrioventricular (AV) and semilunar valves will be evaluated using two-dimensional, color and pulsed Doppler ultrasound. Fetal functional echocardiography will include the following parameters according to published methodology [27]: in the 4-chamber view EDD, ESD, IVS, myocardial wall thicknesses in diastole (calculation of SF and EF for each ventricle) and cardiothoracic ratio. With pulsed Doppler: early and late peaks of transvalvular filling velocities of left and right ventricles (E/A ratio calculation) [28], aortic valve and pulmonary valve peak systolic velocities, velocity time integrals, and fetal heart rate will be measured, and valve diameters will be acquired in 2D for calculation of cardiac outputs. With M-mode: mitral annular-plane systolic excursion (MAPSE) and TAPSE [29]. Finally, left isovolumetric contraction and relaxation times, and ejection time will be measured for calculation of myocardial performance index, as previously described [30].

#### Biomarker analyses

All blood samples will be collected without anticoagulants. Biomarkers sFlt-1, PlGF, hs-cTnT and NT-proBNP will be measured using automated electrochemiluminescence immunoassays on a Cobas E601 platform (Roche Diagnostics GmbH, Mannheim, Germany). Copeptin will be measured in a KRYPTOR lab instrument by an immunoluminometric assay using two polyclonal antibodies (BRAHMS, Thermo-Fischer, Germany )[21]. The clinical laboratory involved in the study performs standard internal and external quality controls for biomarkers included in the study. For interpreting observed values we will use reference values or ranges obtained in previous studies or recommended guidelines.

#### Ambulatory blood pressure monitoring (ABPM)

Blood pressure (BP) measurements will be carried out by a 24-h monitoring with the ABPM 7100 (Welch Allyn, Skaneateles, NY, US) that measures brachial or peripheral BP and central BP, in addition to pulse wave velocity. Peripheral BP will be monitored every 20 min and central BP every 60 min throughout the 24-h period. Day and night periods will be defined according to waking and sleeping periods reported by patients. Peripheral and central BP monitoring will register systolic, diastolic and mean BP during day- and night-time periods and the night/day ratio of the different measurements; peripheral measurements will also include heart rate and pulse wave velocity.

### Data collection

Patients and healthy controls will be asked to complete a questionnaire including demographic, anthropometric, personal and familiar clinical data, with special attention to cardiovascular-related information and PE-related obstetric history (previous PE, fetal growth restriction, abruptio placentae or stillbirth) prior to the examination.

## Outcome measures

### Primary outcome

Maternal cardiac dysfunction or remodeling in the first and third trimesters of pregnancy and 12 months postpartum, defined as any of the following:

- Abnormal cardiac performance in echocardiographic assessments.
- Abnormal concentrations of cardiac biomarkers (Copeptin, hs-cTnT, NT-proBNP).

### Secondary outcomes

- Cardiac dysfunction or remodeling in fetuses from mothers at risk for PE or established PE, defined as abnormal cardiac performance in third trimester echocardiographic ultrasound assessment and/or abnormal cardiac biochemical profile (Copeptin, NT-proBNP, hs-cTnT) in umbilical cord blood at delivery.
- Abnormal BP or lipid profile in the PE group as compared to the others, in the first, third trimester of pregnancy, or at 12 months after delivery.
- Development of a combined model for prediction of PE, including biochemical and biophysical markers of CV and placental dysfunction.
- Cardiac and metabolic status at 12 months after delivery and their correlation with cardiac and PE risk variables obtained during pregnancy. Development of a combined model for prediction of CV risk 12 months after delivery, including biochemical and biophysical markers of CV and placental dysfunction.

## Statistical analysis

### Sample size

We have planned a 3 year-period recruitment, considering the number of deliveries at our institution. To obtain an adequate sample, a total of 2800 screenings must be performed, assuming there is a 1-to-6 ratio between high-risk cases vs controls. We will include all pregnant women attending our center for first trimester screening and agreeing to participate in the study. They will be divided into two groups according to their PE risk; we plan to include 280 pregnancies with high-risk of PE and 100 patients with low-risk of PE, as well as 60 patients with established PE; we have included an estimated 10% drop-rate during the study. The study has already begun the enrollment; as of 30/09/2021 110 high-risk PE patients, 54 low-risk PE patients and 10 established PE patients have been included.

#### Database development and statistical analysis

A web-based database will be developed through a restricted website. All variables will be recorded, and the collected data will be coded and processed with adequate precautions to ensure patient confidentiality. Each participating physician will receive a login name and password to gain access to the web-secured database, with access restricted to clinicians with electronic password.

Once sample collection and laboratory determinations of biomarkers are finished, we will proceed to evaluation and analysis of the results obtained with the aid of the Statistics Department at our institution. Measures will be normalized to expected values for the respective gestational age. For biomarker measurements without reference values in pregnancy, we will obtain reference values from the control population, estimated by linear modeling and describe as multiples of the median (MoM). Variables not following a Gaussian distribution will be log-transformed for analysis.

For the primary outcome, differences in all registered variables among the three study groups will be evaluated at the three evaluation points. Group comparisons will be performed by analysis of variance (ANOVA), followed by post-hoc tests or Chi-squared tests for proportions. Associations and confusion variables will be explored by a multivariate approach, using logistic regression models.

For secondary outcomes, descriptive data will be analyzed at all relevant time-points, frequency of altered values registered and compared among groups and times. Correlations between biochemical markers, including angiogenic imbalance, and cardiac dysfunction/remodeling will be assessed. Diagnostic test parameters (sensitivity, specificity, positive and negative predictive values) will be calculated, and Cox proportional-hazards model will be performed. Univariate and multivariate analysis will allow identifying which variables can be incorporated to a multivariable model to predict CV risk or disease in PE.

In all analyses, a *p* value below 0.05 will be considered statistically significant. Data will be analyzed using the IBM SPSS Statistics 23 statistical package.

#### Quality control and ethics

Study documentation will be saved at least 5 years after completion of the study. Upon request of monitors, auditors, Ethics Committee or health authorities, files related to the study will be available upon request.

## Discussion

In the current study, we hypothesize that in PE, anti-angiogenic imbalance may be associated to maternal and fetal cardiac dysfunction and remodeling, and that both conditions could induce myocardial damage detected by cardiac biomarkers and imaging techniques. Finally, we postulate that such cardiac abnormalities could be predictive of future cardiovascular health in patients with history of PE.

PE is a hypertensive disorder in which predominance of placental anti-angiogenic factors like sFlt-1 over - angiogenic factors like PlGF exists [31]. PE increases 2- to 4-times maternal risk of CVD; although mechanisms linking PE and CVD are not fully understood [32], PlGF and sFlt-1 are regulators of angiogenesis, a mechanism which, if altered, constitutes the basis of several CVD [33].

Pregnancy promotes stressful changes on the maternal CV system, even in normal conditions. Maternal cardiac output and heart rate increase through the three trimesters of pregnancy, while blood pressure and vascular resistances decrease. Both the sympathetic system and renin-angiotensin-aldosterone axis are activated and contribute to an increased circulating volume. At a structural level, enlargement of the four cardiac chambers and an increase of the LV wall mass occur [11]. All these changes are worsened in PE, exceeding normal adaptation. Taken together, CV findings seen in PE resemble cardiac diastolic dysfunction, a condition promoting improper LV relaxation and increasing LV chamber stiffness, resulting in increased cardiac filling pressures [10].

The association between CVD and angiogenic factors has been investigated in non-pregnant populations. Increased concentrations of PlGF at admission are associated with worse outcomes in acute coronary syndromes, although PlGF stimulates angiogenesis and mediates recovery of cardiac pathologies [34]. Increased sFlt-1 concentrations are related to acute coronary occlusion and poor cardiac function in patients with stable coronary disease [35], and with development of endothelial vascular dysfunction, myocardial injury and adverse CV outcomes in patients with congestive heart failure [35]. As in PE, the use of the sFlt-1/PlGF ratio appears to be more predictive of adverse outcomes in coronary artery disease than either biomarker alone [36]. Finally, although angiogenic factors are critical players in PE endothelial dysfunction, there is no evidence supporting their role as maternal cardiotoxic factors, and CV risk later in life. A possible explanation could be that maternal cardiac damage in PE is so subtle that it cannot be detected by current imaging techniques; therefore, the use of cardiac biomarkers may have a role for early detection.

Some studies have shown that hs-cTn is elevated in pregnant women with hypertension or with PE, compared with healthy pregnancies. These are strong predictors for development of both conditions [37] and are related to mean peak arterial BP in asymptomatic PE patients [38]. Knowing hs-cTn can detect minor myocardial damage like that existing due to ischemia, we shall assess whether PE causes such damage. In addition to hs-cTn, copeptin and natriuretic peptides could be helpful biomarkers for cardiac dysfunction in PE. Copeptin concentrations may be increased in PE, because it is not only a regulator of circulating volume, but also responsive to the CV stress associated with the condition [39]. Volume changes and hemodynamic stress are characteristics of pregnancy that are particularly strong in PE. Santillan [20] was the first to report a high sensitivity and specificity of copeptin to predict PE in the first and second trimesters of pregnancy. A recent meta-analysis has confirmed these findings, extending them to the 3rd trimester, and showing a correlation between copeptin concentrations and PE severity [40]. On other hand, secretion of natriuretic peptides is increased in response to increases in BP, cardiac filling volume, cardiac chamber pressure and dilation [32]; consequently, natriuretic peptide concentrations are increased in PE [41]. We believe NT-proBNP concentrations in pregnant women may be an earlier and alternative tool to echocardiography for detecting cardiac function alterations existing in PE. Our study plans to use all information generated by these biomarkers, along with clinical and echocardiographic variables, to develop a CV risk score during a preeclamptic pregnancy, and at 12 months after delivery.

Studies conducted in patients with heart failure and in patients with PE have found a possible common pathophysiological pathway mediated by angiogenic/anti-angiogenic placental markers like PIGF and sFlt-1 [42, 43]. Our group has recently shown that 12 years after delivery, lower levels of PlGF in pregnancy affected by PE were are associated with worse maternal hemodynamics (higher BP) and lower high-density lipoprotein cholesterol concentrations and with subclinical myocardial dysfunction (worse global longitudinal strain) and increased carotid intima-media thickness [16]. The results of this study corroborate that angiogenic imbalance in pregnancies complicated by PE is related to a negative impact in future CV risk, long term after pregnancy. These findings compel to further study causes of PE and the possibility of prediction and prevention. In the present study, we wish to assess if an anti-angiogenic profile from the beginning of pregnancy determines CV remodeling, that in turn, promotes PE development and associated CV injury that can persist after pregnancy, conditioning future CV health.

In addition to maternal assessment, we plan to evaluate fetal cardiac function at the third trimester of pregnancy, to analyze if there are potential associations between maternal CV status and angiogenic factors and occurrence of fetal cardiac programming. Recently, various studies have demonstrated the occurrence of a fetal cardiac programming in the growth-restricted fetus, which persists into childhood [19]. Furthermore, this fetal cardiac remodeling is also present in an independent fashion, in pregnancies affected by preeclampsia [18]. Given that fetal growth-restriction and PE are clinical expressions of underlying placental disease, we hypothesize and plan to analyze whether angiogenic factors will be associated with the existence of cardiac remodeling.

There are potential limitations and considerations of the present project. We would only observe subclinical CV changes due to the short length of postpartum follow-up (12 months). Clinically evident CV outcomes will probably require much longer follow-up given that the studied population will only include young, pregnant women. However, our aim is to initiate a cohort study of patients with high-risk for PE or that develop the disease, in order to adequately follow-up in the long-term, as we have previously done with other cohorts. Development of a predictive model of future CVD risk or complications, including clinical and echocardiographic variables, CV and placental biomarkers, will require further validation in prospective well-powered studies. Finally, in our study we will assess associations between CV findings and angiogenic factors but finding of potential causal links between both will still require further studies.

To conclude, this study will provide, for the first time, evidence characterizing the association between angiogenic imbalance and CV dysfunction/remodeling in women at risk for or developing PE and, in their offspring, both during and after pregnancy. We expect to provide data supporting the association between abnormal angiogenesis and CV dysfunction/remodeling during pregnancy and fetal life, therefore identifying new therapeutic targets for future studies aiming to prevent CV morbidity associated with PE. The goal of this research is to obtain insight in the particular risk factors that link pregnancy and CV health, and thus, reduce the burden of the disease.

## Data Availability

Datasets that will be used and analyzed for the presented study will be available from the corresponding author on reasonable request; they will not be publicly available due to patient confidentiality.

## Abbreviations

ABPM: Ambulatory Blood Pressure Monitoring
AV: Atrioventricular
BP: Blood pressure
CV: Cardiovascular
CVD: Cardiovascular disease
EDD: End-diastolic diameter
EDV: End-diastolic volume
EF: Ejection fraction
ESD: End-systolic diameter
ESV: End-systolic volume
HR: Heart rate
hs-cTnT: High-sensitivity cardiac troponin T
IVS: Interventricular septum
LV: Left ventricle
LVOT: Left ventricle outflow tract
MAPSE: Mitral annular-ring systolic excursion
NT-proBNP: N-terminal portion of the proBNP natriuretic peptide
PAPP-A: Pregnancy-associated plasma protein A
PlGF: Placental growth factor
PCR: Polymerase chain reaction
PE: Preeclampsia
PW: Posterior wall
sEng: Soluble endoglin
sFlt-1: Soluble fms-like tyrosine kinase 1
SF: Shortening fraction
TAPSE: Tricuspid annular-ring systolic excursion
TDI: Tissue Doppler imaging
VEGF: Vascular endothelial growth factor
UtA-PI: Uterine artery pulsatility index
